# Lockdown COVID‑19: a stress test revealing more about the mental health system than psychopathology—A comparative analysis between Austria, Germany, and Italy

**DOI:** 10.3389/fpubh.2026.1516371

**Published:** 2026-04-01

**Authors:** Francesco de Bertoldi, Livio Finos, Pascale Roux, Davide Gaspari, Jan Di Pauli, Heiko Ullrich, Nicolas Benedetti, Jacopo Bizzarri, Johanna Neugebauer, P. Peter Plum, Mariateresa Nitti, Andreas Conca

**Affiliations:** 1 Department of Psychiatry, General Hospital of Bolzano, Sanitary Agency of South Tyrol, Bolzano, Italy; 2 Department of Statistical Sciences, University of Padova, Padova, Italy; 3 Vorarlberg University of Applied Sciences, Empirical Social Sciences Research Group, Dornbirn, Austria; 4 Regional Hospital of Rankweil, Adult Psychiatry, Rankweil, Austria; 5 Department of Psychiatry, Psychotherapy and Psychosomatics, Siegen Hospital, Siegen, Germany; 6 Geriatrics Department, General Hospital of Bolzano, Sanitary Agency of South Tyrol, Bolzano, Italy

**Keywords:** COVID-19, health policy management, psychopathology, quarantine, temporal analysis, transnational study

## Abstract

**Background:**

This study examined whether the COVID-19 pandemic produced diagnosis-specific and cross-nationally reproducible alterations in psychiatric admissions, or whether observed variations primarily reflected organizational responses. We compared psychiatric inpatient services from Austria and Germany—two hospital-centered systems with similar pre-pandemic structures—and used Italy as a contrasting community-based model.

**Methods:**

We conducted a retrospective multicenter observational study including all psychiatric hospitalizations between 2017 and 2020. Temporal admission trends were analyzed bi-weekly for each ICD-10 diagnostic group. Generalized Linear Models (Poisson family) were used to model the effects of Diagnosis, Year (2020 vs. pre-2020), Week, and Country, and their interactions. Diagnostic categories were grouped following ICD-10, with F10 (alcohol-related disorders) analyzed separately from other substance-use disorders to capture differences between planned and emergency admissions.

**Results:**

Across Austria and Germany, 2020 was associated with a marked temporal distortion in admission patterns compared with previous years (Week × Year interaction, *p* < 0.05), consistent across diagnoses except for F10. Alcohol-related disorders showed country-specific trajectories, reflecting service reorganization—bed closures in Austria versus stable capacity in Germany. No other diagnosis exhibited reproducible temporal deviations across countries, indicating that the pandemic affected admission organization rather than disorder-specific psychopathology. A supplementary comparison including Italy showed that, while Austria and Germany experienced a decline in total hospitalizations (−16 and −10%, respectively), Italy remained stable (+12%), suggesting greater resilience of its community-based system.

**Conclusion:**

The pandemic did not induce diagnosis-specific increases in acute psychiatric admissions but substantially altered their temporal organization. Differences across countries highlight how healthcare structure—particularly the integration of territorial and inpatient services—modulates system resilience under crisis conditions.

## Introduction

The quarantine imposed by governments during the first year of the COVID-19 pandemic undeniably succeeded in halting the spread of the infection (1). However, these benefits must be balanced against potential psychological repercussions on the population (2, 3). Recent evidence indicates that anxiety and depressive symptoms increased globally during the pandemic, although with considerable heterogeneity across populations and time frames (4, 5). Previous studies have reported a high prevalence of anxiety, depression, post-traumatic stress, and aggression in populations subjected to quarantine during past pandemics (2), as well as a greater risk of mental health deterioration among psychiatric patients compared with the general population (6). Social distancing, fear, and economic instability may have acted as stressors for individuals with anxiety or mood-related disorders (7–9). Conversely, several large-scale reviews suggest that the rise in self-reported symptoms did not uniformly translate into higher rates of diagnosed mental disorders or suicide, emphasizing the influence of contextual and organizational factors (10). Cultural factors have also been shown to shape cognitive and behavioral responses, particularly under conditions of uncertainty (11). To better understand the effects of quarantine on distinct subtypes of psychiatric disorders, several studies have analyzed diagnostic distributions among psychiatric emergency visits and hospital admissions during the pandemic, often by comparing them with equivalent pre-pandemic periods (12). However, findings have been inconsistent—reporting decreases in psychotic or mood disorders (13), increases (14), or no significant variation (15). From a theoretical standpoint, it has been hypothesized that quarantine may exert a protective effect on severe mental disorders, based on the idea that the psychopathological core of psychosis lies in the interaction between the self and the external world (16). Beyond methodological heterogeneity, interpretation is complicated by multiple concurrent influences on hospitalization during lockdowns—clinical, political (infection-control policies), organizational (availability of psychiatric beds), and cultural or social factors. Evidence from Austria and Germany shows substantial service reorganizations during lockdowns, including altered admission thresholds and emergency pathways, suggesting that healthcare policies may have shaped hospitalization trends independently of psychopathology (17, 18). In summary, the impact of quarantine on different subtypes of psychiatric disorders remains a controversial issue, particularly in terms of its implications for understanding underlying psychopathological mechanisms. To address this question, an informative strategy involves comparing psychiatric services across countries. Such comparisons can help identify both shared and divergent patterns of hospitalization under identical pandemic restrictions but differing healthcare systems. We hypothesize that, if quarantine exerted a specific protective or aggravating effect on certain diagnostic groups, this effect should be observable across different national settings—since quarantine represents the constant environmental factor in the comparison. To explore this hypothesis, we focused on the entire first pandemic year, capturing both the initial and subsequent lockdowns, using a fine-grained analytical approach based on bi-weekly hospitalization trends for each diagnostic category. Diagnostic groups were classified according to the ICD-10 system (F0–F9). To ensure adequate sample size and statistical stability, categories F5 through F9—representing behavioral, personality, developmental, and childhood-onset disorders—were aggregated into a single group. Conversely, within the substance-related domain (F1), we applied a more granular subdivision by separating alcohol-related disorders (F10) from other psychoactive substance-use disorders. This decision was based on clinical and organizational considerations: while alcohol-related admissions are frequently planned or programmatically regulated (e.g., detoxification and rehabilitation), other substance-use admissions are typically acute and unscheduled. This separation allowed us to assess more precisely how pandemic-related disruptions differentially affected scheduled versus emergency pathways of care.

The objective of this study is therefore to determine—through a direct comparison between two countries with broadly comparable psychiatric service structures (Austria and Germany)—whether fluctuations in psychiatric admissions during quarantine reflect primarily psychopathological changes or rather organizational responses to crisis conditions. Additionally, data from an Italian service are presented for descriptive contrast, providing an example of how a distinct community-based model may display different hospitalization dynamics under the same global constraints.

## Methods

### Study design

A retrospective multicenter transnational observational study was conducted on patients admitted to SPDC during the first year of the pandemic. We involved three psychiatric services located in three different countries with similar socio-demographic settings by comparing hospitalization rate time-trends for each diagnostic group. The hospitalization trends observed in 2020 were evaluated in comparison to the pre-2020 period (average admission rate of 2017, 2018, and 2019).

### Participating centers

- Germany: Kreisklinikum Siegen GmbH—140 beds for a region of 280,000 inhabitants. Each year, 2,300 patients are treated.
- Austria: Regional Hospital of Rankweil, Psychiatry—150 beds for a region of 370,000 inhabitants. Each year, 2,400 patients are treated.
- Contrasting model—Italy: Hospital of Bolzano, Department of Psychiatry—26 beds for a region of 240,000 inhabitants. Each year, 700 patients are treated.

### Time frame considered

January 1st to December 31st of years 2017–2018–2019–2020.

### Study population

A comprehensive survey of all patients admitted to the SPDC during the study period was conducted in all three participating institutions.

**Figure 1:**
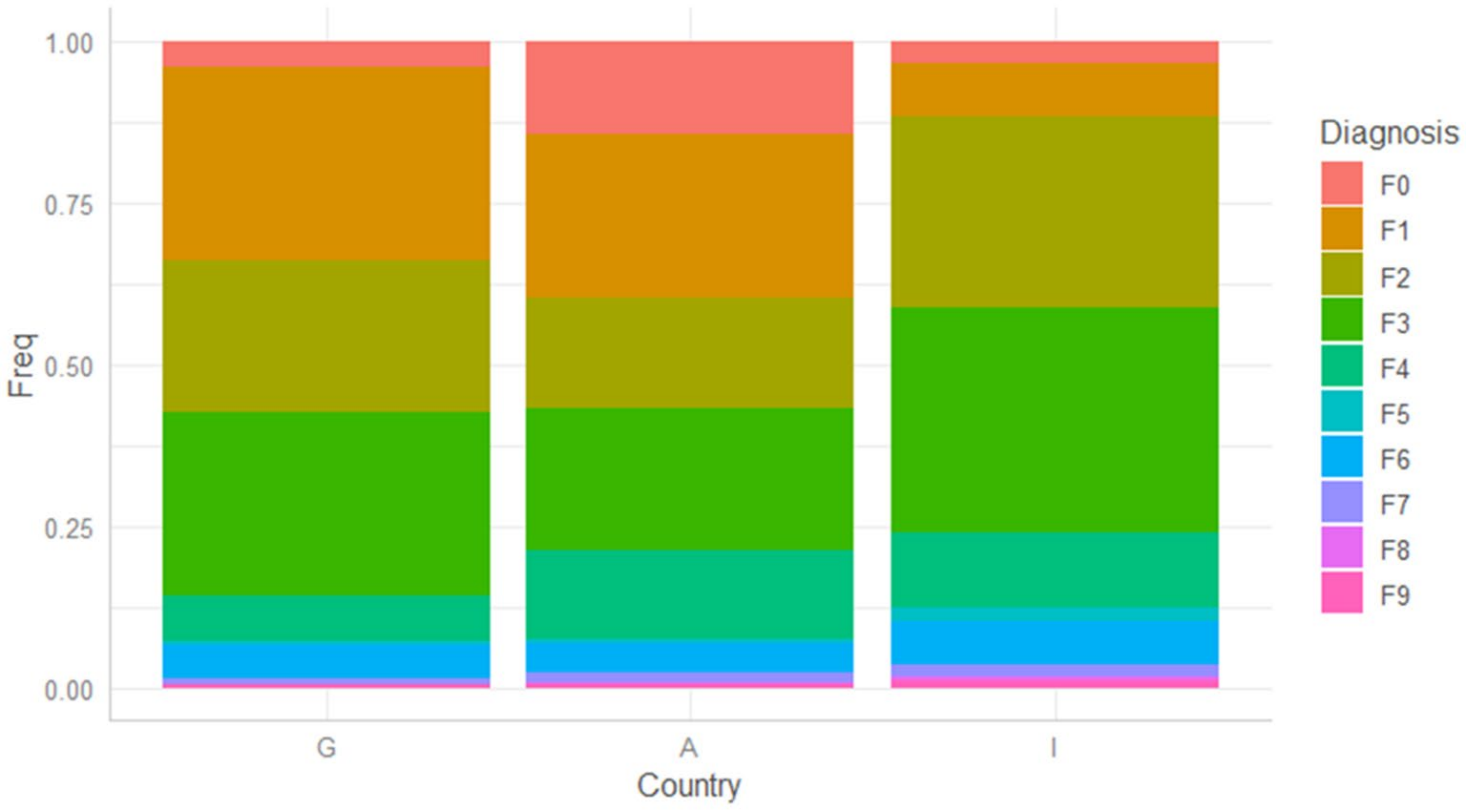
Distribution by diagnostic group of patients for each psychiatric service.

Average age (Table 1), distribution of admissions by gender (Table 2, Figure 1), and by diagnosis (Table 3) are shown for each psychiatric service.

**Table 1:** Average age of patients for each psychiatric service.

Age	M		F	
	Mean	SD	Mean	SD
G	51.0041	18.824	45.92298	17.73677
A	52.14502	19.969	48.03057	19.18216
I	47.41057	17.584	43.0154	15.99887

**Table 2:** Distribution by gender of patients for each psychiatric service.

	M	W	Total
G	0.531	0.469	1.000
A	0.519	0.481	1.000
I	0.503	0.497	1.000

**Table 3:** Analysis of Siegen (Germany).

Term	Df	Deviance	Resid. Df	Resid. Dev	Pr(>Chi)
NULL	NA	NA	375	6332.187	NA
Diagn	6	3118.689	369	3213.498	0.000
ns(Week, df = 4)	4	71.864	365	3141.635	0.000
Year	1	2624.889	364	516.745	0.000
Diagn:ns(Week, df = 4)	24	37.112	340	479.633	0.043
Diagn:Year	6	21.887	334	457.747	0.001
ns(Week, df = 4):Year	4	10.600	330	447.147	0.031
Diagn:ns(Week, df = 4):Year	24	24.908	306	422.239	0.411

Signif. codes: 0, ‘***’ p<0.001, ‘**’ *p*<0.01, ‘*’ *p*<0.05, ‘.’ *p*<0.1, ‘*’ *p* ≥0.1.

Inclusion Criteria: Any gender and ethnic background aged 18 or older.

Exclusion Criteria: Patients admitted as part of the so-called “revolving door phenomenon” (readmission within one month) were excluded, as these phenomena develop independently of the COVID-19 pandemic (see Table 4).

**Table 4:** Analysis of Rankweil (Austria).

Term	Df	Deviance	Resid. Df	Resid. Dev	Pr(>Chi)
NULL	NA	NA	376	5013.010	NA
Diagn	6	1179.408	370	3833.602	0.000
ns(Week, df = 4)	4	53.794	366	3779.808	0.000
Year	1	3132.585	365	647.223	0.000
Diagn:ns(Week, df = 4)	24	24.108	341	623.115	0.455
Diagn:Year	6	28.396	335	594.719	0.000
ns(Week, df = 4):Year	4	11.867	331	582.852	0.018
Diagn:ns(Week, df = 4):Year	24	48.593	307	534.259	0.002

Signif. codes: 0, ‘***’ p<0.001, ‘**’ *p*<0.01, ‘*’ *p*<0.05, ‘.’ *p*<0.1, ‘*’ *p* ≥0.1.

### Diagnostic classification

Diagnoses were grouped according to the ICD-10 classification system into the following macro-categories:

- F0: Organic, including symptomatic mental disorders
- F1: Mental and behavioral disorders due to use of psychoactive substances
- F10: Alcohol-related disorders (analyzed separately due to programmatic, scheduled admissions)
- F2: Schizophrenia, schizotypal, and delusional disorders
- F3: Mood [affective] disorders
- F4: Neurotic, stress-related, and somatoform disorders
- F5–F9: Aggregated into a single group to ensure sufficient sample size (behavioral, personality, developmental, and childhood-onset disorders)

### Main analysis (Austria–Germany comparison)

#### Statistical method

A Generalized Linear Model (GLM) with Poisson response (counts) was adopted to model four variables (33):

(a) Diagnosis: the number of admissions for each diagnostic group (categorical variable).
(b) Year: 2020 (pandemic onset) vs. pre-2020 (sum of the three previous years, categorical variable).
(c) Bi-weekly variation: number of admissions in a two-week time window (quantitative variable).
(d) Country: the analyzed services (categorical variable).

Nonlinear dynamics across weeks were modeled using a Natural Cubic Splines basis with 4 degrees of freedom (df) (34). The model included all main effects and two-, three-, and four-way interactions. The main research question was whether the temporal dynamics of Week changed during the pandemic period compared to previous years (Year). If the Week × Year test proved significant, additional within-diagnosis models were run to verify for which diagnostic categories the effect was confirmed. Significance was corrected using the Bonferroni-Holm procedure (35). All analyses were conducted with R software (19).

### Phase 1—within-country modeling

For Austria and Germany, separate Poisson GLMs were fitted to examine the effects of Year (pre-2020 vs. 2020), nonlinear temporal dynamics (Week, modeled with splines), Diagnosis, and their interactions.

For each diagnostic category, a separate within-country model was estimated to test the Week × Year interaction.

### Phase 2—between-country comparative modeling

A combined GLM including the factor Country was used to assess differences between Austria and Germany in baseline diagnostic composition and temporal changes from pre-2020 to 2020.

Analyses were repeated using standard ICD-10 groupings with a finer scheme separating alcohol-related disorders (F10) from the rest of the F1 category (other substances).

### Visual descriptive observations

As an initial descriptive step, we performed visual descriptive observations to assess whether the COVID-19 pandemic altered the temporal structure of psychiatric hospitalizations across countries and diagnostic categories.

**Figure 2:**
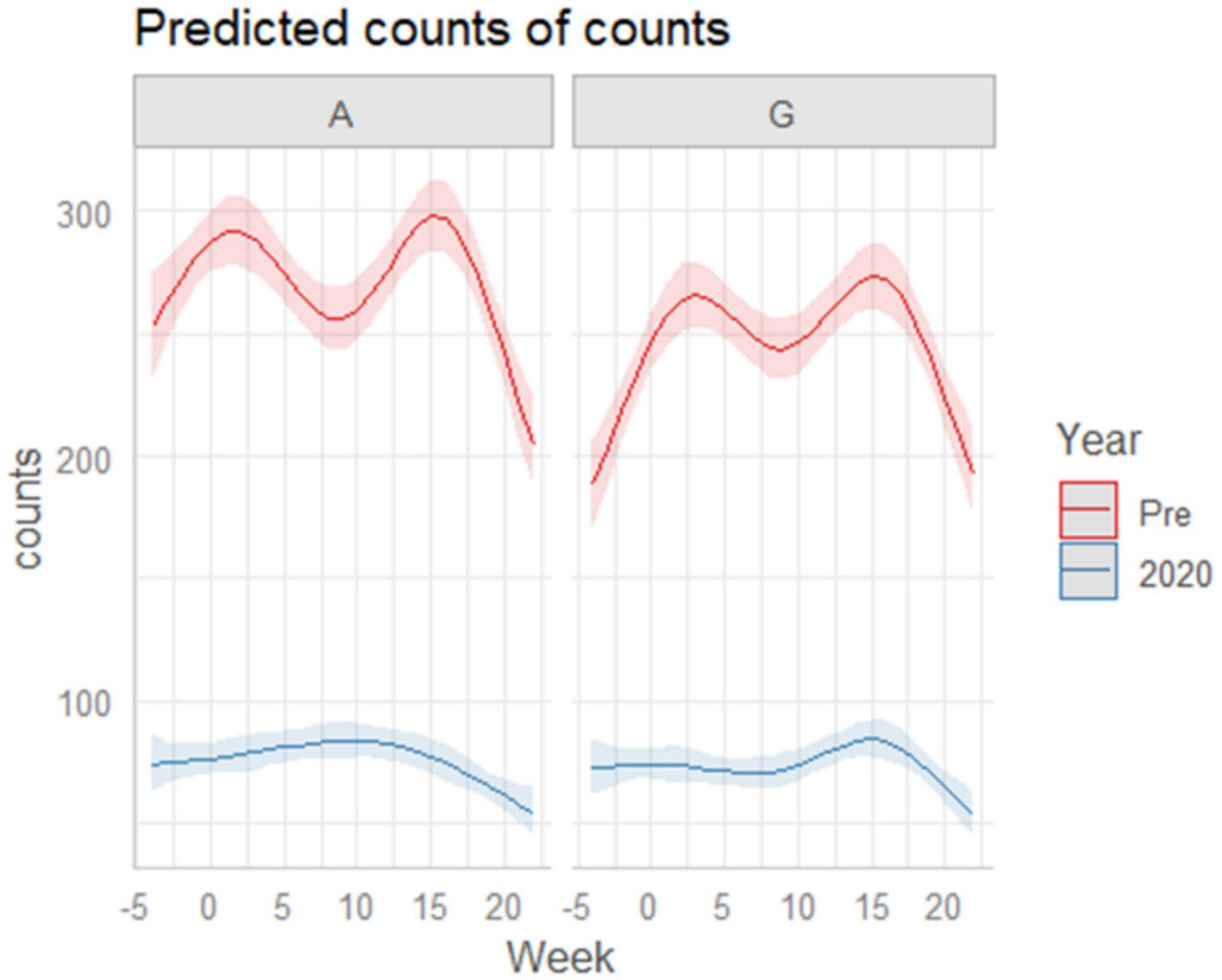
Predicted bi-weekly hospitalization trends for Austria, Germany, and Italy in the pre-pandemic period (2017–2019) and during 2020. Curves represent the smoothed temporal trajectories generated from the Poisson GLM using natural cubic splines (4 df), averaged across all ICD-10 diagnostic categories. In the pre-2020 period, Austria and Germany display highly comparable biphasic patterns with a spring peak (around April) and an autumn peak (around October). In 2020, both countries show marked temporal distortions associated with the first pandemic lockdown: in Germany the typical spring peak disappears entirely, while in Austria the biphasic structure is lost and the curve flattens, with only a modest rise during the summer months. Italy, shown for descriptive context, exhibits substantially lower hospitalization numbers, producing flatter curves and smaller differences between pre-2020 and 2020 trends. The reduced baseline volume reflects its predominantly community-based psychiatric system and serves to illustrate magnitude differences across services rather than to inform cross-country inferential comparisons.

**Figure 3:**
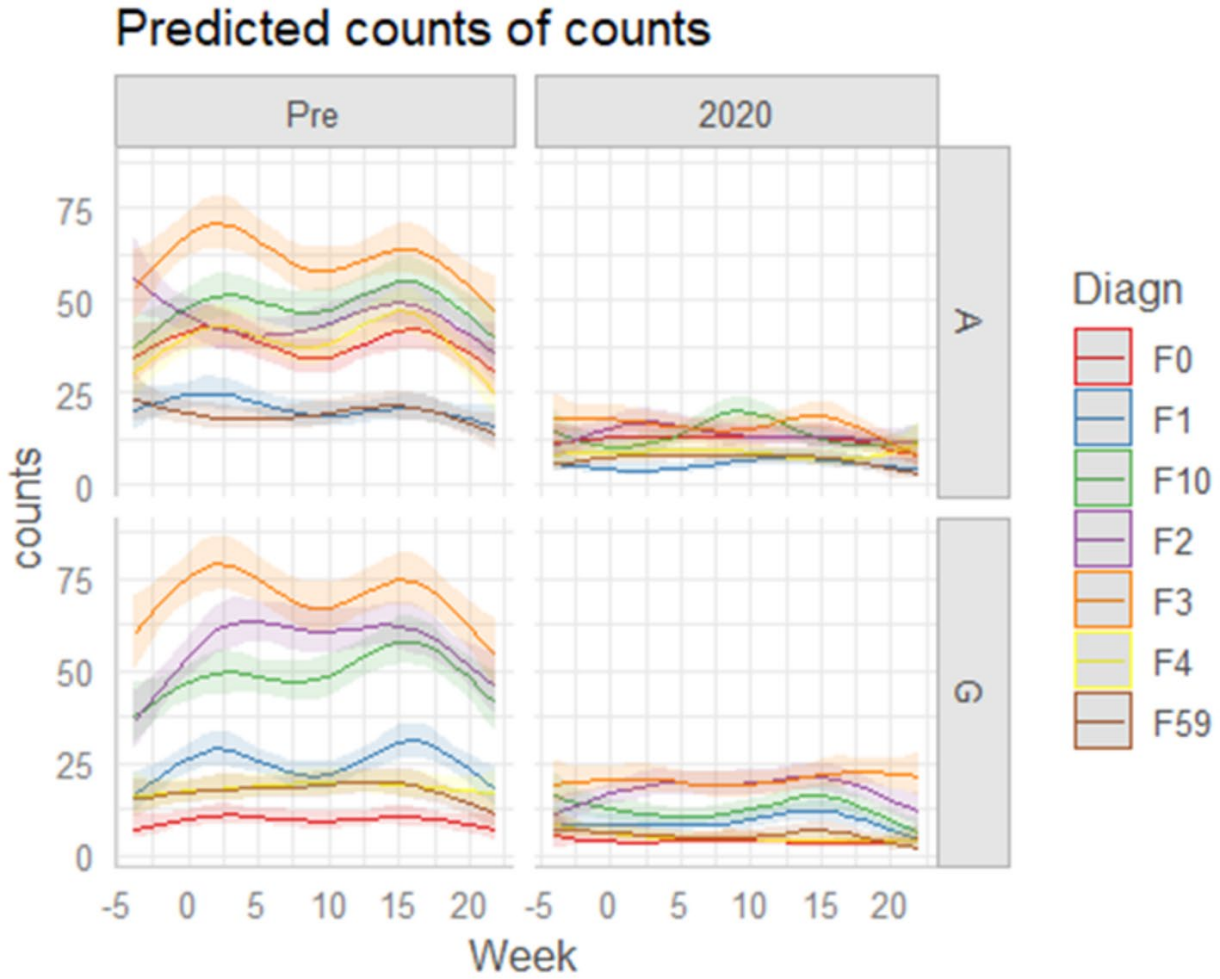
Predicted bi-weekly hospitalization trends for Austria (left panels) and Germany (right panels) obtained from the Poisson Generalized Linear Model (GLM) with natural cubic splines. Each diagnostic category is represented by a separate smoothed curve, and alcohol-related disorders (F10) are modeled independently from other substance-use disorders (F1). Upper panels display the pre-pandemic reference period (2017–2019), while lower panels show the corresponding trajectories for 2020. In the pre-2020 period, both countries exhibit stable and regular diagnostic time-courses, with modest fluctuations and no marked divergence between diagnostic groups. In 2020, however, lockdown-related disruptions introduce clear alterations. In Germany, diagnosis-specific curves show a relatively homogeneous pattern characterized by the disappearance of the spring peak and a shared increase around the second lockdown period in November. In Austria, temporal deviations are more heterogeneous and diagnosis-dependent. Alcohol-related disorders (F10) show a distinctive inverted biphasic profile, with reductions during both lockdown periods and a pronounced rebound during the summer months (around August). Other diagnostic groups display diverse distortions relative to pre-2020 patterns, highlighting cross-country differences in the temporal reorganization of inpatient psychiatric care during the pandemic year.

**Figure 4:**
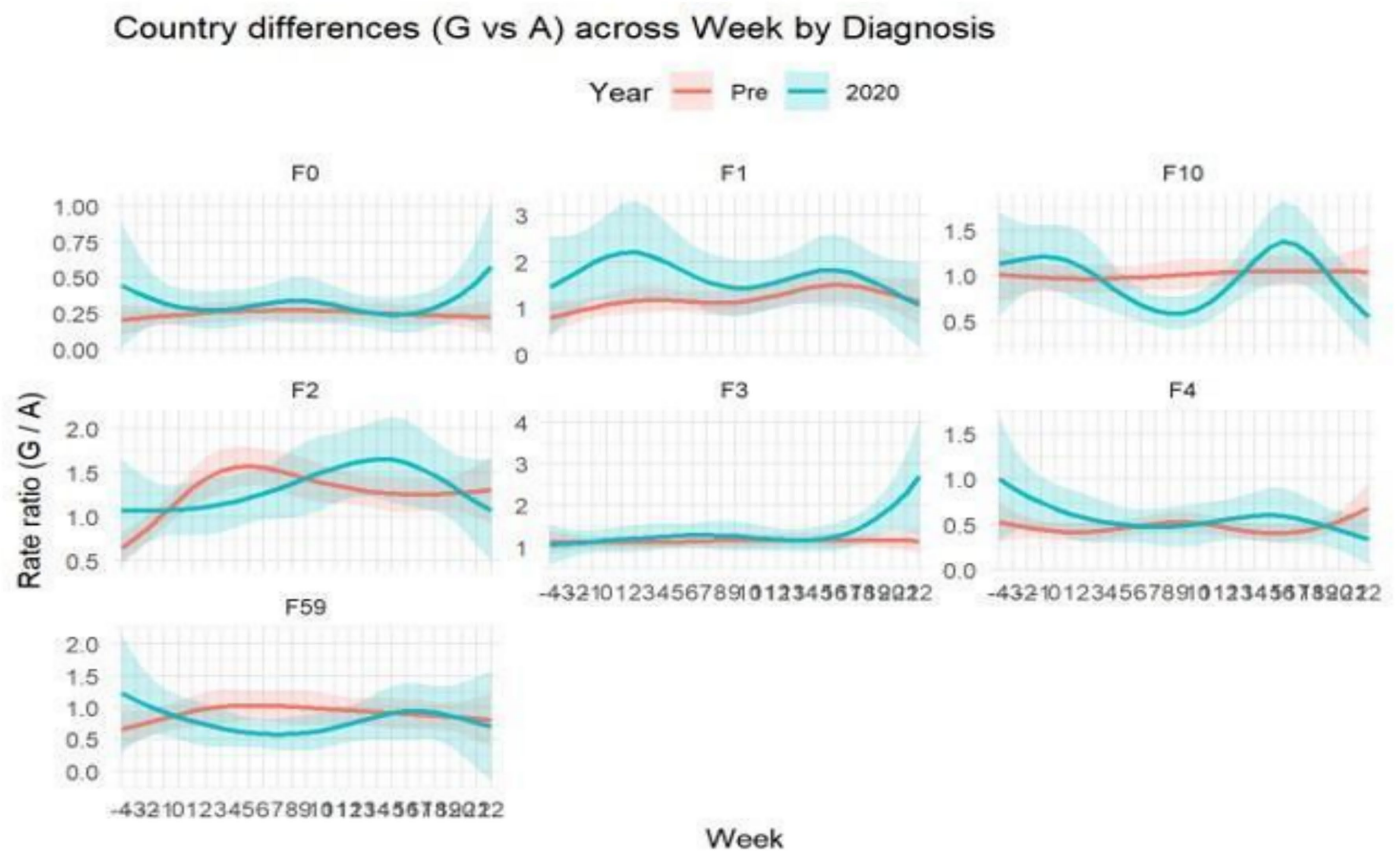
The lines represent the ratio of admissions in G to those in A for each diagnostic category. A value of 1 indicates no variation between the two countries, while values greater than 1 mean higher admissions in Germany and values less than 1 mean higher admissions in Austria. A flat horizontal line indicates proportionality between the two countries. These ratios appear very similar for all diagnoses before the COVID-19 pandemic (red lines), whereas non-flat lines are visible during the COVID-19 period (cyan lines), revealing different patterns between the two countries. From the analyses and visual inspection, as can be seen, the red lines tend to be more linear throughout the year, showing that differences between the two countries remained stable during the 3 years preceding the pandemic, while in 2020 more marked differences emerged, particularly for F10. We considered as indicative of significance the temporal phases showing distinct patterns between Germany and Austria, with no significant overlap within the standard deviation area of the two time-trend curves.

1 Temporal alignment and construction of time-trend plots (Figures 2–4)

Hospitalization counts were aggregated in 15-day intervals for each country. To ensure temporal comparability, Week 0 was set on 8 March 2020, corresponding to the first national lockdown in Italy (the first lockdown in Europe). Based on this reference point, two shared lockdown windows were defined:

First lockdown (time frames 0–5)

Italy: 8 March—23 May

Austria: 16 March—13 April

Germany: 23 March—10 May.

Second lockdown (time frames 15–17)

Italy: 23 October—30 November

Austria: 11 November—30 November

Germany: 2 November—30 November.

For each diagnostic category, the 2020 trajectory was visually compared with the mean pre-pandemic trend (2017–2019).

2 Diagnosis-specific visual inspection (Figure 3)

To explore whether temporal deviations differed across diagnostic groups, we generated diagnosis-specific trajectories for Austria and Germany based on the predicted values of the within-country Poisson GLMs (described in the Statistical Method section).

This allowed us to visually assess whether:

- temporal distortions in 2020 were uniform across diagnoses, or
- whether diagnosis-specific deviations emerged,
- and whether these patterns differed between the two hospital-based systems.

3 Between-country temporal ratio analysis (Figure 4)

As an additional step, we plotted the weekly Germany/Austria ratio of admissions for each diagnostic category.

- A value of 1 indicated proportionality between the countries
- Values >1 indicated higher admissions in Germany
- Values <1 indicated higher admissions in Austria

In the pre-2020 period, stable horizontal ratios were interpreted as reflecting consistent cross-country proportionality for that diagnosis (even if absolute volumes differed).

Deviations observed in 2020 were taken as visual indicators of pandemic-related divergence in system behavior.

Integrative role of visual descriptive observations.

These descriptive procedures provided a framework that guided but did not determine the inferential analyses. The quantitative modeling presented in the subsequent sections formally tested whether the qualitative patterns observed here corresponded to statistically significant diagnosis-specific or country-specific temporal effects.

### Supplementary analysis (cross-country comparison including Italy)

To explore the influence of different healthcare system structures, a supplementary analysis compared Austria and Germany (hospital-based systems) with Italy (a community-based model).

We quantified the variation in total admissions in 2020 relative to the mean of the three preceding years (2017–2019).

To ensure comparability across countries, diagnostic categories F0 (organic and symptomatic disorders) and F1/F10 (substance- and alcohol-related disorders) were excluded, given substantial differences in structure and treatment organization across healthcare systems.

## Results

### Main analysis (Austria–Germany comparison)

#### Phase 1—within-country modeling

Germany.

### Germany (Siegen)

The analysis revealed significant main effects of diagnosis, year, and bi-weekly temporal variation (Week), all showing highly significant deviations (*p* < 0.001). The interaction between diagnosis and Week was also significant (*p* = 0.043), indicating modest differences in the temporal structure of hospitalizations across diagnostic categories. The Year × Week interaction was significant as well (*p* = 0.031), confirming that the overall temporal distribution of admissions in 2020 differed from that of the preceding 3 years. A significant Diagnosis × Year interaction (*p* = 0.001) further indicated that the relative diagnostic composition of admissions shifted during the pandemic year. In contrast, the three-way interaction Diagnosis × Week × Year was not significant (*p* = 0.411), suggesting that, although hospitalization timing changed in 2020, this deviation occurred homogeneously across diagnostic groups. Overall, the German sample exhibited a broad reorganization of temporal patterns and diagnostic composition during the pandemic year, but without evidence of diagnosis-specific temporal signatures.

### Austria

The analysis revealed significant main effects of diagnosis, year, and bi-weekly temporal variation, each showing highly significant deviations (*p* < 0.001). The Year × Week interaction was also significant (*p* = 0.018), confirming that the temporal distribution of hospitalizations in 2020 differed from that observed across the previous 3 years. Importantly, both the Diagnosis × Year interaction (*p* < 0.001) and the Diagnosis × Week × Year interaction (*p* = 0.002) were significant. These findings indicate that the pandemic year introduced diagnosis-specific temporal fluctuations that were not present in the pre-2020 period. In contrast, the Diagnosis × Week interaction alone was not significant (*p* = 0.455), suggesting that temporal differences among diagnostic groups emerged only in relation to the pandemic context, rather than reflecting stable pre-existing differences across diagnoses. In summary, the Austrian dataset shows a marked diagnosis-specific reorganization of inpatient admissions during 2020, with heterogeneous and diagnosis-dependent temporal trajectories—in clear contrast to the more homogeneous temporal response observed in Germany.

### Phase 2—between-country comparative modeling

#### Visual descriptive observations

##### Descriptive analysis of global hospitalization trends

We first examined the global temporal organization of hospitalizations across Austria and Germany during the pre-pandemic years and in 2020 (Figure 2). In the pre-2020 period, both countries displayed highly comparable trajectories characterized by a clear biphasic seasonal pattern, with a spring peak (around April) and an autumn peak (around October). In 2020, this organization was markedly disrupted following the introduction of the first lockdown. In Germany, the spring peak consistently observed in previous years disappeared almost entirely, indicating a pronounced suppression of admissions during the early lockdown phase, whereas the autumn peak remained visible. In Austria, the 2020 curve lost its typical biphasic shape and followed a flattened trajectory, with only a modest increase during the summer months (around August). These descriptive trends illustrate a shared pandemic-related distortion in the temporal structure of hospitalizations across the two hospital-based psychiatric systems.

### Diagnosis-specific temporal patterns in Austria and Germany

To determine whether the temporal alterations observed at the aggregate level also emerged within specific diagnostic categories, we examined the predicted trajectories from the Poisson GLMs for Austria and Germany separately (Figure 3). In the pre-2020 period, both countries exhibited smooth and consistent temporal trajectories across diagnostic categories. In 2020, however, lockdown-related disruptions produced visible and heterogeneous distortions, with distinct profiles in Austria and Germany. In Germany, most diagnostic categories showed a relatively homogeneous response: the disappearance of the spring peak was observed across diagnoses, along with a shared increase around November, corresponding to the second lockdown. In Austria, temporal deviations were more pronounced and clearly diagnosis-specific. Several diagnostic groups diverged substantially from their pre-2020 trajectories, indicating a less uniform systemic response. Alcohol-related disorders (F10) displayed the most distinctive pattern, characterized by an inverted biphasic trajectory—a marked reduction during both lockdowns followed by a rebound in summer (around August). Other diagnostic groups exhibited heterogeneous alterations, reflecting a stronger diagnosis-specific reorganization of inpatient care. Together, these findings suggest that the temporal reorganization of psychiatric admissions during 2020 varied both by diagnosis and by healthcare system. This provides a first exploratory visual evidence—later confirmed by the quantitative analyses—that the impact of the pandemic on inpatient psychiatric care was not uniform but shaped by diagnosis-specific and system-level factors.

### Between-country comparative modelling (Austria vs. Germany)

To quantify diagnostic-specific differences between Austria and Germany and their evolution over time, we modelled the weekly ratio of admissions (Germany/Austria) for each ICD-10 diagnostic category (Figure 4). A value of 1 indicates parity between the two countries, values above 1 indicate higher admission rates in Germany, and values below 1 indicate higher rates in Austria. In the pre-pandemic years (red lines), the curves remained largely stable over time, but not necessarily centered on 1. This indicates that, although the seasonal trajectories were comparable across the two countries, the absolute hospitalization levels differed systematically for certain diagnoses—for instance, F0 showed stable ratios around 0.25, meaning that Germany consistently admitted about half as many patients as Austria, despite similar temporal patterns. In contrast, during 2020 (cyan lines), several diagnostic categories exhibited marked temporal deviations, reflecting system-specific alterations under pandemic conditions. The most evident divergence was observed for alcohol-related disorders (F10), where Germany showed a relative increase in admissions compared to Austria during the summer and autumn months, suggesting differences in how each system reorganized addiction-related care during the pandemic. Other diagnostic categories displayed subtler but still noticeable shifts, indicating that the pandemic altered the long-standing equilibrium between the two systems. Overall, these findings show that although Austria and Germany shared similar pre-pandemic temporal organizations of psychiatric care, the pandemic year introduced new asymmetries, particularly for diagnostic categories sensitive to service accessibility and treatment organization, such as substance- and alcohol-related disorders (Figure 4).

### Quantitative analyses

#### General effects: country, diagnosis, and year

In the Austria–Germany model, both Country and Diagnosis showed significant main effects, confirming baseline differences in admission volumes and diagnostic composition between the two systems (Table 5). As expected, Year was also highly significant, indicating a global reduction in psychiatric hospitalizations in 2020 compared to the pre-pandemic years.

**Table 5:** Analysis of deviance—Full Austria–Germany model: results of the generalized linear model including country, diagnosis, year (2020 vs. pre-2020), nonlinear temporal effects (week, natural cubic splines with 4 df), and all interaction terms.

Term	Df	Deviance	Resid. Df	Resid. Dev
Country	1	42.79	375	<0.001***
ns(Week, df = 4)	4	53.68	371	<0.001***
Year	1	39.96	370	<0.001***
Diagnosis	6	1179.41	364	<0.001***
Country: ns(Week, df = 4)	4	4.72	360	0.03*
Country:Year	1	2.58	359	0.09.
ns(Week, df = 4):Year	4	9.45	355	0.05*
Country:Diagnosis	6	148.73	349	<0.001***
ns(Week, df = 4):Diagnosis	24	24.11	325	ns
Year:Diagnosis	6	28.40	319	<0.001***
Country: ns(Week, df = 4):Year	4	6.47	315	0.04*
Country: ns(Week, df = 4):Diagnosis	24	5.92	291	ns
Country:Year:Diagnosis	6	9.57	285	0.02*
ns(Week, df = 4):Year:Diagnosis	24	3.53	261	ns
Country: ns(Week, df = 4):Year:Diagnosis	24	3.17	237	ns

Signif. codes: 0, ‘***’ p<0.001, ‘**’ *p*<0.01, ‘*’ *p*<0.05, ‘.’ *p*<0.1, ‘*’ *p* ≥0.1.

#### Temporal effects (Week, Week × Year)

The model revealed a robust Week × Year interaction (Table 5), demonstrating that the temporal distribution of admissions in 2020 deviated markedly from the pre-2020 pattern. This reflects a general system-level distortion in hospitalization timing during the first pandemic year.

The three-way Diagnosis × Week × Year interaction did not reach significance, suggesting that this temporal perturbation occurred broadly across diagnostic categories without strong evidence of diagnosis-specific profiles. However, to explore diagnosis-level effects more precisely, we conducted a set of diagnosis-specific models testing the Week × Year interaction separately for each ICD-10 group. Raw and Holm-corrected *p*-values are reported in Table 6.

**Table 6:** Diagnosis-specific test of the ns(Week, 4) × Year interaction ANOVA results for each ICD-10 diagnostic group.

Diagn	N_obs	ChiSq	DF	*p*_value	*p*_value_Holm
F0	106	3.241	4	0.5183	1.0000
F1	108	11.358	4	0.0228	0.1368
**F10**	**108**	**15.798**	**4**	**0.0033**	**0.0231**
F2	108	4.520	4	0.3402	1.0000
F3	108	3.629	4	0.4585	1.0000
F4	108	9.453	4	0.0507	0.2535
F59	107	3.179	4	0.5283	1.0000

Raw and Holm-adjusted *p*-values are reported.

#### Between-country temporal comparison (Austria vs. Germany)

To determine whether the pandemic-related temporal changes differed between the two healthcare systems, we examined the Country × Week × Year interaction for each diagnostic category. As shown in Table 7, this interaction was significant only for F10 (alcohol-related disorders), and non-significant for all other diagnoses.

**Table 7:** Diagnosis-specific test of the Country × Week × Year interaction ANOVA results assessing whether 2020 temporal distortions differed between Austria and Germany.

Diagn	N_obs	ChiSq	DF	*p*_value	*p*_value_adjusted
F0	106	3.167	4	0.5302	1.0000
F1	108	3.304	4	0.5082	1.0000
**F10**	**108**	**11.158**	**4**	**0.0248**	**0.1736**
F2	108	7.040	4	0.1338	0.7056
F3	108	7.369	4	0.1176	0.7056
F4	108	6.470	4	0.1667	0.7056
F59	107	5.916	4	0.2055	0.7056

Holm-adjusted *p*-values are reported. Bold values indicate statistically significant results after Holm correction (adjusted *p* < 0.05).

This indicates that:

- F1, F2, F3, F4, and F59 showed similar pandemic-related temporal distortions in both countries, with no evidence of cross-national divergence.
- F10 displayed significantly different 2020 trajectories in Austria and Germany, concentrated in a mid-summer interval. This is consistent with system-specific reorganizations of addiction-related inpatient pathways.

Finally, the four-way Diagnosis × Week × Year × Country interaction was not significant (Table 5), indicating no broad diagnostic-specific cross-national temporal patterns once all factors were considered simultaneously.

#### Higher-order interactions

Finally, the four-way interaction Diagnosis × Week × Year × Country was not significant, not allowing to claim any diagnostic-specific temporal signatures across the two countries once all factors were considered simultaneously.

#### Supplementary analysis (cross-country comparison including Italy)

**Figure 5:**
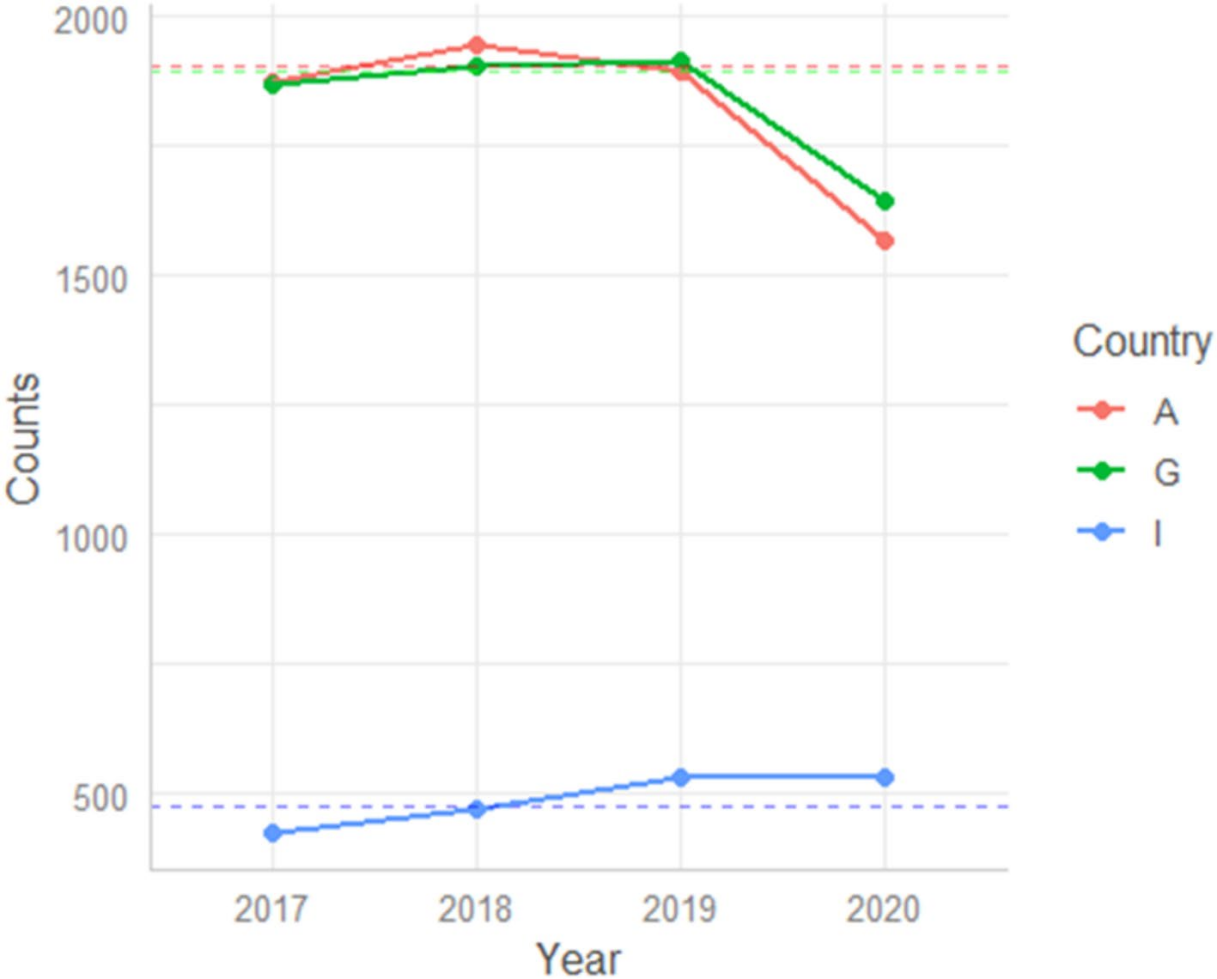
Annual psychiatric hospitalization trends for Austria (red line), Germany (green line), and Italy (blue line) from 2017 to 2020. Lines represent total admissions per year, averaged across all ICD-10 categories except F0 and F1. Dotted horizontal lines indicate the pre-pandemic baseline (2017–2019 mean). While Austria and Germany display a pronounced drop in 2020, the Italian curve remains stable, reflecting the resilience of its community-based psychiatric model.

To explore whether the pandemic-related reduction in hospitalizations observed in Austria and Germany also extended to different healthcare models, we conducted a supplementary analysis including the Italian center as a contrasting case. As detailed in the Methods section, diagnostic groups F0 (organic disorders) and F1 (substance-related disorders) were excluded from this comparison, as these categories are not routinely hospitalized in Italy due to its community-based psychiatric system. When considering the remaining diagnostic categories, both Austria and Germany showed a marked reduction in overall psychiatric admissions in 2020 compared with the mean of the three preceding years (−16.2% and −10.3%, respectively). In contrast, Italy exhibited a slight increase in hospitalizations (+12.1%) (Table 8, Figure 5).

**Table 8:** Cross-country comparison of total psychiatric admissions (excluding F0 and F1 diagnostic categories) between the pre-pandemic period (mean of 2017–2019) and 2020.

	Pre	Average 2017, 2018, 2019	2020	Difference	Variation
A	7,299	2433.00	2039	394.00	−16.19%
G	6,612	2204.00	1978	226.00	−10.25%
I	1,427	475.67	533	−57.33	+12.05%

Austria and Germany both showed a marked decrease in admissions (−16.2% and −10.3%, respectively), whereas Italy exhibited a modest increase (+12.1%). Values represent total counts, mean annual pre-pandemic admissions, absolute difference, and percentage variation relative to the pre-pandemic mean.

## Discussion

This study investigated whether the COVID-19 pandemic produced diagnosis-specific and cross-nationally reproducible alterations in psychiatric admission patterns. Our analyses were intentionally centered on Austria and Germany, two hospital-based psychiatric systems with comparable organizational structures and closely aligned pre-pandemic admission trends. Italy, which follows a distinct community-based psychiatric model with markedly lower hospitalization rates, was included only for descriptive contextualization and not for inferential comparison (14, 20). Across both Austria and Germany, we observed that the year 2020 introduced a substantial distortion in the weekly distribution of psychiatric admissions compared with 2017–2019. This aligns with broader evidence showing that the pandemic disrupted access to mental health services worldwide (10, 12, 18, 21–24). In our data, this temporal alteration was present across all major ICD-10 diagnostic groups. However, the magnitude of the change did not differ significantly between diagnoses, indicating that no diagnostic category was selectively increased or reduced relative to others during the pandemic—a finding consistent with large-scale reports showing heterogeneous symptom changes but limited evidence for diagnosis-specific incidence shifts (4, 5, 10). Because substance-related disorders encompass both scheduled and unscheduled pathways of care, we separated alcohol-related disorders (F10) from other substance-use disorders (F1). This distinction is supported by clinical and organizational considerations: admissions for alcohol dependence are frequently planned or embedded in structured detoxification and rehabilitation programs (25–28), whereas F1 presentations predominantly reflect acute, unplanned emergencies. Hence, scheduled pathways are more sensitive to system-level disruptions, and separating F10 and F1 allowed us to more precisely evaluate how the pandemic influenced distinct care trajectories within the domain of addiction. When Austria and Germany were modeled together, a clear pattern appeared. For all diagnostic groups except F10, temporal changes observed in 2020 showed a similar structure in both countries. This suggests that the pandemic induced a broad organizational perturbation—such as modified admission thresholds, reduced service availability, or pathway reorganization—rather than diagnosis-specific psychopathological effects. This interpretation is consistent with prior studies indicating that service-level changes, rather than direct psychopathological mechanisms, often drive fluctuations in psychiatric hospital utilization during crises (12, 15, 18, 22). F10 was the only category showing different temporal trajectories between Austria and Germany. This divergence mirrored differences in service organization. In Austria, the 80 inpatient beds dedicated to alcohol-related disorders were temporarily closed during the first lockdown and remained partially unavailable during the second wave, whereas Germany maintained full operative capacity for addiction-related admissions. These decisions explain the sharp lockdown-related decline and subsequent summer rebound for F10 in Austria, and the relative stability observed in Germany. A phenomenon present in only one country cannot plausibly be attributed to a psychopathological mechanism; instead, it reflects organizational effects tied to system-specific constraints. Taken together, our results indicate that the pandemic produced a widespread reorganization of psychiatric service utilization but did not generate diagnosis-specific or cross-nationally reproducible temporal signatures suggestive of a direct quarantine-psychopathology effect. Rather, the patterns align with organizational responses to crisis conditions, including interruptions to structured treatment pathways, modified admission criteria, and temporary reduction of bed capacity (12, 13, 18, 21). To further explore how systemic organization may moderate the impact of global crises on psychiatric care, we performed an additional cross-country comparison including Italy as a contrasting model. When excluding diagnostic categories F0 and F1 to ensure comparability, Austria and Germany both showed a clear reduction in total inpatient admissions in 2020 (−16.2% and −10.3%, respectively), whereas Italy exhibited remarkable stability (+12.1%). This stability may reflect structural features of the Italian mental health system, which has long been organized according to a community-based and territorially integrated model (29, 30). Because Italian psychiatric wards primarily admit acute and severe cases, hospitalization thresholds were already high before the pandemic; consequently, the COVID-19 emergency did not substantially alter inpatient volume (31). At the same time, Italian Community Mental Health Centers (CSM) likely absorbed part of the clinical demand, maintaining continuity of care even under restrictive conditions (32, 36, 37). In contrast, Austria and Germany—whose systems remain more hospital-centered—experienced sharper contractions in inpatient activity, likely due to temporary ward reorganization, reduced bed availability, and stricter admission thresholds (17, 18). Overall, these findings suggest that the Italian system may have demonstrated greater resilience and adaptability in maintaining psychiatric care continuity during the pandemic (27, 28). This study indicates that no diagnosis-specific effects of social isolation emerged in terms of acute symptom exacerbation leading to hospitalization. Instead, the pandemic exerted a strong influence on the organization and temporal dynamics of admissions, highlighting the structural vulnerability of hospital-centered systems under crisis conditions.

### Limitations

This study has limitations. The cross-national comparison involved two hospital centers rather than national datasets, and larger multicenter cohorts may reveal more nuanced diagnosis-specific effects. Larger samples would also allow more granular analyses, especially within substance-related disorders, where patterns may differ across subgroups (26–28). Future studies should incorporate demographic variables, socioeconomic indicators, and measures of lockdown stringency, which have been shown to modulate mental health outcomes during the pandemic (1–5). Although formal stringency indices report that Italy implemented slightly stricter measures during the first lockdown, Austria and Germany followed a broadly comparable two-phase pattern of restrictions (spring and autumn 2020). Given our biweekly temporal resolution and the organizational focus of our analysis, these moderate differences in restriction intensity were unlikely to introduce systematic bias, which is why no explicit “stringency” covariate was included. Extending analyses beyond 2020 will help determine whether these disruptions persisted or gradually normalized.

### Conclusions and future perspectives

Despite these limitations, the analytic framework applied here—integrating diagnostic, temporal, and cross-national dimensions—offers a model for understanding how psychiatric systems respond to acute external disruptions. Our findings highlight the importance of resilient organizational planning, the maintenance of structured care pathways, and flexible resource allocation during public health emergencies. Diagnostic groups relying on planned admissions, such as alcohol-related disorders, may require particular attention to ensure continuity of care during future crises (25–28). Future research should extend this approach to post-pandemic longitudinal outcomes and broader service metrics to assess whether the apparent resilience of community-based systems translates into sustained mental-health benefits and more equitable access to care.

## Data Availability

The original contributions presented in the study are included in the article/supplementary material, further inquiries can be directed to the corresponding author.
